# Hip Fracture Post-operative Mortality and Polypharmacy: A New Risk Predictor?

**DOI:** 10.7759/cureus.47089

**Published:** 2023-10-15

**Authors:** Yousef Al-Khatib, Kishore Dasari

**Affiliations:** 1 Trauma and Orthopaedics, George Eliot Hospital, Nuneaton, GBR

**Keywords:** hip hemiarthroplasty, antegrade intramedullary nail, dynamic hip screw fixation, polypharmacy, nottingham hip fracture score, elderly hip fractures

## Abstract

Introduction

Hip fractures include intra- and extracapsular fractures with hip hemiarthroplasty, intramedullary (IM nailing), dynamic hip screw (DHS) fixation and cannulated cancellous screws being the main treatment options. The Nottingham Hip Fracture Score (NHFS) is used to predict the risk of 30-day mortality with some studies investigating its use for one-year mortality. This study aims to investigate the impact of polypharmacy on post-operative hip fracture mortality and the correlation with NHFS predicted mortality.

Methods

A retrospective single-centre analysis was carried out on hip fracture patients aged 65 years and over who underwent operative management. Primary outcome measures were 30-day and one-year mortality along with the presence of polypharmacy. Secondary outcome measures were mortality based on procedure type, NHFSs for polypharmacy and non-polypharmacy groups, number of medications, American Society of Anesthesiologists (ASA) grade, age and gender. Polypharmacy was defined as five or more long-term medications from a selected list of drug classes.

Results

Thirty-day mortality was 19.3% for the polypharmacy group and 2.4% for the non-polypharmacy group (P≤0.00001), while one year mortality was 50.9% for the polypharmacy group and 2.4% for the non-polypharmacy group (P≤0.00001), the NHFS was 5.16 (±1.38) on average for the polypharmacy group and 5.07 (±1.47) for the non-polypharmacy group. Thirty-day mortality was 10/116 (8.6%) for the hemiarthroplasty patients, 3/66 (4.5%) for the DHS fixation patients and 1/32 (4.5%) for the IM nailing patients. One-year mortality was 33/116 (28.4%) for the hemiarthroplasty patients, 11/66 (16.7%) for the DHS fixation patients and 4/32 (12.6%) for the IM nailing patients.

Conclusion

Polypharmacy correlated with a significantly higher one-year and 30-day postoperative mortality after hip fractures with the NHFS predicting no difference in mortality. This finding could assist in decision making and help facilitate discussions with patients and family members regarding post-operative mortality risks. The NHFS may also benefit from integrating polypharmacy possibly leading to more accurate risk predictions. The IM nailing and DHS fixation patients were found to have a lower 30-day and one-year mortality than the hemiarthroplasty patients.

## Introduction

Hip fractures include intra- and extracapsular fractures [1,2]; these fractures are treated using various methods, including dynamic hip screw (DHS) fixation, intramedullary (IM) nailing, hemiarthroplasty, total hip arthroplasty and cannulated cancellous screws [1]. Hip fractures have a mortality of 20-30% on average within one year and 6-12% on average within 30 days [3-5]. Frailty, old age and multiple comorbidities along with cognitive impairment have been associated with an increased risk of death after hip fractures [6].

The Nottingham Hip Fracture Score (NHFS) is a score used to estimate mortality risk at 30 days using the above-mentioned risk factors, such as age, comorbidities and other factors [6,7]. Studies have also investigated the use of the NHFS to predict one-year mortality, where a score of over five was associated with a high risk of mortality at one year [8]. Polypharmacy is most commonly defined as five or more regular long-term medications [9]. Anti-hypertensives amongst other drug classes were associated with the highest hospital readmissions [10]. Our study aims to demonstrate the association of polypharmacy with higher mortality rates and the correlation between polypharmacy and NHFS-predicted mortality.

## Materials and methods

A single-centre retrospective analysis of hip fracture patients aged 65 or over who underwent operative fixation of a hip fracture between the first of January 2021 and the 31st of December 2021 was performed. The primary outcome measure was 30-day and one-year mortality along with the presence of polypharmacy. Secondary outcome measures were mortality based on the procedure type and NHFSs for both groups.

We considered a patient to have polypharmacy if they were on five or more regular, long-term (>6 months) medications out of a particular list of drug classes [9,10]. The number of medications rather than drug classes was counted. Combination drugs were counted as one medication.

Our list of drug classes included anti-platelets, diuretics, non-steroidal anti-inflammatory drugs, anticoagulants, opioids, beta-blockers, drugs affecting the renin-angiotensin system, drugs used in diabetes, positive ionotropes, antidepressants, calcium channel blockers, anti-epileptics, nitrates, inhaled corticosteroids, potassium channel activators and anti-asthmatics [10].

Two hundred and twenty-four patients were identified as hip fracture patients who underwent surgical treatments. Patient records were reviewed to record the operation type, polypharmacy status, number of medications, one-year mortality, 30-day mortality, NHFSs, American Society of Anesthesiologists (ASA) grade, age and gender. An average NHFS was calculated for both the polypharmacy and non-polypharmacy groups to help standardise the 30-day mortality risk. Mortality rates were then calculated for each operation type.

Statistical analysis (Microsoft Excel version 21, Microsoft, USA) was then applied where a standard deviation for the NHFS and the number of medications was calculated. Chi-squared testing was applied, and P-values were calculated for the 30-day and one-year mortality in the polypharmacy group compared to the non-polypharmacy group, with significance defined as P<0.05. Logistic regression analysis was then applied for the ASA grade, NHFS and number of medications to investigate their impact on both the 30-day and one-year mortality.

## Results

**Figure 1:**
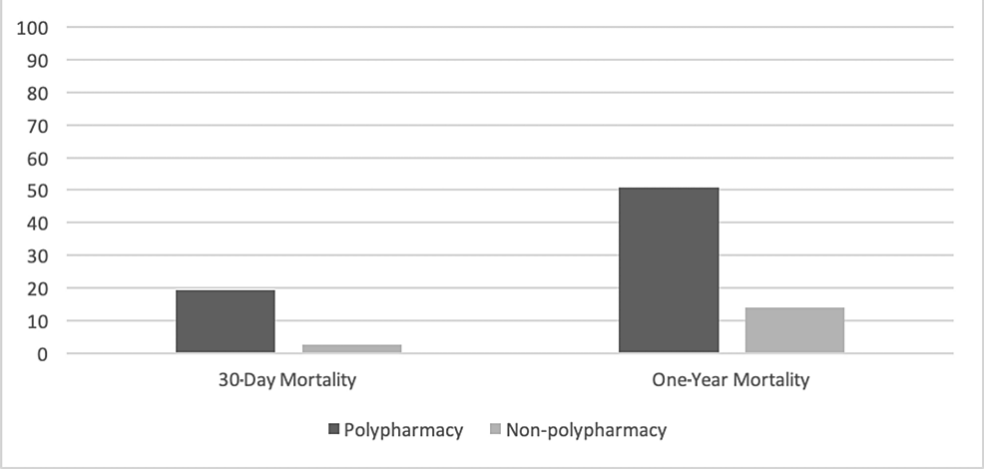
Percentage of the one-year mortality and 30-day mortality of the polypharmacy group compared to the non-polypharmacy group. Data are presented as %.

A total of 224 patients were identified with the total one-year mortality being 52/224 (23.21%) and the total 30-day mortality being 15/224 (6.7%). Fifty-seven of the 224 (25.4%) patients were found to have polypharmacy, of which 29 (50.9%) died within one year, while 167/224 (74.6%) of the patients did not have polypharmacy, of which 23 (13.8%) died within a year (P≤0.00001). With regard to the 30-day mortality, the polypharmacy group had 11 deaths (19.3%) compared to four (2.4%) deaths in the non-polypharmacy group (P≤0.00001) (Figure 1). The NHFS was 5.16 (±1.38) on average for the polypharmacy group and 5.07 (±1.47) for the non-polypharmacy group.

The average number of medications was three medications overall; the non-polypharmacy group had an average of 2.04 medications while the polypharmacy group had an average of 5.75 medications. Patients who died within one year had an average of 4.24 medications, and those who died within 30 days had an average of 4.93 medications. Patients with a survival of over one year had an average of 2.67 medications. Logistic regression found a P-value of less than 0.05 for the number of medications and 30-day and one-year mortality (Tables 1, 2). Odds ratio (OR) was 1.79 for the 30-day mortality and 1.50 for the one-year mortality (Tables 1, 2). ASA grade was found to have a P-value of 0.15 for the 30-day mortality and and 0.22 for the one-year mortality (OR=2.32) for 30-day mortality and 1.62 for one-year mortality (Tables 1, 2). Logistic regression also found a P-value of 0.0144 for the NHFS and 30-day mortality and 0.0006 for one-year mortality (Tables 1, 2). The NHFS had an OR of 1.69 for 30-day mortality and 1.57 for one-year mortality (Tables 1, 2).

**Table 1:** Logistic regression analysis comparing the ASA grade, NHFS and number of medications and 30-day mortality. Data are presented as coefficient, standard error, P-value, odds ratio and 95% confidence interval. Significance is defined as P<0.05. ASA: American Society of Anesthesiologists; NHFS: Nottingham Hip Fracture Score

Variable	Coefficient	Standard error	P-value	Odds ratio	95% confidence interval
Number of medications	0.5797	0.1746	0.0009	1.7855	(1.2681, 2.5138)
NHFS	0.5261	0.2149	0.0144	1.6923	(1.1106, 2.5786)
ASA grade	0.8401	0.5879	0.1530	2.3167	(0.7319, 7.3332)

**Table 2:** Logistic regression analysis comparing the ASA grade, NHFS and number of medications and one-year mortality. Data are presented as coefficient, standard error, P-value, odds ratio and 95% confidence interval. Significance is defined as P<0.05. ASA: American Society of Anesthesiologists; NHFS: Nottingham Hip Fracture Score

Variable	Coefficient	Standard error	P-value	Odds ratio	95% confidence interval
Number of medications	0.4040	0.0951	0.0000	1.4978	(1.2430, 1.8048)
NHFS	0.4511	0.1307	0.0006	1.5701	(1.2152, 2.0286)
ASA grade	0.4851	0.3933	0.2174	1.6243	(0.7514, 3.5113)

The hemiarthroplasty patients had a one-year mortality of 33/116 (28.4%) and a 30-day mortality of 10/116 (8.6%). DHS fixation patients had a one-year mortality of 11/66 (16.7%) and a 30-day mortality of 3/66 (4.5%). IM nailing had a one-year mortality of 4/32 (12.5%) and a 30-day mortality of 1/32 (3.26%). Cannulated screw fixation had a one-year mortality of 4/10 (40%) and a 30-day mortality of 1/10 (10%) (Table 3). 

**Table 3:** One-year and 30-day mortality based on the procedure type. Data are presented as N and %.

Procedure	Number of patients	One-year mortality	30-day mortality
Hemiarthroplasty	116	33 (28.4%)	10 (8.6%)
Dynamic hip screw fixation	66	11 (16.7%)	3 (4.5%)
Intramedullary nailing	32	4 (12.5%)	1 (3.26%)
Cannulated screw fixation	10	4 (40%)	1 (10%)

With regard to age and gender, there were 152 females and 72 males with an average age of 81.74 years (±9.08). The polypharmacy group had an average age of 81.3 years (±8.5), and the non-polypharmacy group had an average age of 81.85 years (±7.61).

## Discussion

Our most significant finding was the one-year and 30-day mortality being higher in the polypharmacy group compared to the non-polypharmacy group despite the NHFS predicting a very similar mortality for both groups. For both groups (polypharmacy and non-polypharmacy), the 30-day mortality would be 6.9-11.8% according to the updated NHFS [11]. Our study found a lower 30-day mortality rate than predicted for the non-polypharmacy group and a higher rate for the non-polypharmacy group. The average NHFS for both groups being very similar means that a possible consideration should be made for polypharmacy to be incorporated into the NHFS or for polypharmacy to be taken into account in risk prediction for patients prior to surgery. Our P-values further demonstrate the significance of the correlation found between polypharmacy and 30-day and one-year mortality. The NHFS does take comorbidities into consideration, which may not always correlate with polypharmacy as some patients may be on five or more medications for hypertension, which would count as a single comorbidity, giving the patient no extra points on the NHFS as it does not meet the minimum of two comorbidities. 

The average number of medications for the >one-year survival group was 2.67, just over half the number we considered as polypharmacy. This shows the impact of polypharmacy on mortality when compared to an average of 4.24 medications for the one-year mortality group and 4.93 for the 30-day mortality group. Logistic regression analysis also found a significant correlation between the number of medications and 30-day (OR= 1.79) and one-year mortality (OR= 1.5). Further studies investigating the use of the NHFS with polypharmacy integrated would be suggested as it could lead to more accurate mortality risk prediction. ASA grading was found to have no statistically significant correlation with the one-year (P=0.2174) or 30-day mortality (P=0.153) according to the logistic regression analysis. There was however a significant correlation between the NHFS and 30-day and one-year mortality. This is consistent with current literature reports stating that the NHFS is a more accurate mortality predictor than the ASA grade [7]. Studies have discussed the impact of polypharmacy on the likelihood and risk of neck of femur fractures [12]. Another multicentre study has found that polypharmacy is associated with higher post-operative mortality [13]. Our data compare the risk of mortality as predicted by the NHFS to the percentage risk with and without polypharmacy. Frailty, age, gender, comorbidities, cognition, low haemoglobin and care home residence are factors associated with an increased mortality risk post femur neck fracture surgical fixation [6]. These risk factors are all taken into consideration in the NHFS; polypharmacy or the number of medications is not.

With regard to the procedure type, it was found that hemiarthroplasty had a higher one-year and 30-day mortality than DHS fixation and IM nailing. This is consistent with reports in the literature on 30-day mortality [14]. One-year mortality was reported to be similar (28% for hemiarthroplasty and 29% for internal fixation), while this study reports a lower rate for DHS fixation and IM nailing when compared to the hemiarthroplasty cohort [14]. Cannulated screw fixation patients had the highest mortality rate (4/10, 40% one-year mortality). This was consistent with reports in the literature that report a post-operative mortality of 50% in one year for cannulated cancellous screw fixation patients [15].

The female population was over two times that of the male population in this study. This is expected as elderly women are at a higher risk of osteoporosis [16], with osteoporosis being a predisposing factor to hip fractures [17]. Our average age was also found to be high, with no clear difference in the average age between the polypharmacy and non-polypharmacy groups. This is also expected as the elderly population is most prone to hip fractures [18].

In terms of weaknesses to this study, the retrospective design could have led to selection bias. Cannulated cancellous screw and groups were small with only 10 patients. Patients who were managed conservatively were also not included in this study with most of the patients being unfit for surgery, which would have changed our mortality rates if included. Including conservatively managed patients would have likely increased our average mortality rate as they are reported to have a relatively high (74% 30-day and 92% one-year mortality) mortality rate compared to operatively managed patients [19].

## Conclusions

Polypharmacy was associated with a higher 30-day and one-year mortality rates in hip fracture patients with the NHFS predicting almost no difference in mortality. This can assist in the decision making and help facilitate discussions with patients and their families when informing them of their post-operative mortality risk. IM nailing operations having the lowest 30-day and one-year mortality with cancellous screw fixation being the highest could further assist in clinical decision making along with family discussions. Future studies could potentially investigate if the NHFS provides more accurate predictions of 30-day and one-year mortality with polypharmacy integrated and investigate the impact of polypharmacy on mortality based on the procedure type.
